# Prevalence and factors related to urinary incontinence in older adults women worldwide: a comprehensive systematic review and meta-analysis of observational studies

**DOI:** 10.1186/s12877-021-02135-8

**Published:** 2021-03-29

**Authors:** Sedighe Batmani, Rostam Jalali, Masoud Mohammadi, Shadi Bokaee

**Affiliations:** 1 Department of Nursing, School of Nursing and Midwifery, Kermanshah University of Medical Sciences, Kermanshah, Iran; 2 Faculty of Health and Life Sciences, School of Life Sciences, Coventry University, Coventry, UK

**Keywords:** Prevalence, Urinary incontinence, Women, Older adults, Meta-analysis

## Abstract

**Background:**

Urinary incontinence is a common condition in the general population and, in particular, the older adults population, which reduces the quality of life of these people, so this study aims to systematically examine and meta-analyse the overall prevalence of urinary incontinence in older women around the world and the related and influential factors.

**Methods:**

This report is a comprehensive systematic review and meta-analysis of the findings of research on urinary incontinence in older adults people across the world through looking for MEDLINE, Cochrane Library Sciencedirect, Embase, Scopus, ProQuest and Persian databases, namely iranmedex, magiran, and SID from January 2000 to April 2020, the heterogeneity of the experiments was measured using the I^2^ index and the data processing was done in the Systematic Meta-Analysis programme.

**Results:**

In 29 studies and the sample size of 518,465 people in the age range of 55–106 years, urinary incontinence in older adults’ women in the world based on a meta-analysis of 37.1% (95% CI: 29.6–45.4%) was obtained. The highest prevalence of urinary incontinence was reported in older adults’ women in Asia with 45.1% (95% CI: 36.9–53.5%). Meta-regression also showed that with increasing the sample size and year of the study, the overall prevalence of urinary incontinence in the older adults women of the world decreased and increased, respectively, which were statistically significant differences (*P* <  0.05). According to studies, the most important factors influencing the incidence of urinary incontinence in older women are women’s age (*p* <  0.001), obesity (p <  0.001), diabetes (p <  0.001), women’s education (p <  0.001), delivery rank (p <  0.001), hypertension (p <  0.001), smoking (p <  0.001). They also have urinary tract infections (p <  0.001).

**Conclusion:**

Given the high prevalence of urinary incontinence in older women around the world, health policy makers must consider control and diagnostic measures in older women and prioritize treatment and rehabilitation activities.

## Background

The World Health Organization (WHO) finds citizens 65 years of age to be older adults and the United Nations deems people with 60 years or above to be older adults [1, 2]. The world’s population is aging rapidly, with 703 million people now over the age of 65, and this number is projected to reach 1.5 billion by 2050 [3]. Urinary incontinence is a common condition in the general population, especially the older adults, which reduces the quality of life so that ten to 20 % of all women and 77% of women living in nursing homes have urinary incontinence [4]. According to the International Association of Urinary Incontinence (ICS), any involuntary leakage of urine is called urinary incontinence (UI) [5].

Urinary incontinence is divided into three categories: stress, urgency and combination. Stress urinary incontinence (SUI) refers to the leakage of urine due to increased intra-abdominal pressure such as exercise and cough, which is due to the poor functional urethra. In connection with the reduction of anatomical support due to trauma, vaginal delivery, obesity and increased intra-abdominal pressure due to chronic constipation, lifting heavy objects and exercise is called urinary excretion with or above the distance after the sensation of excretion, urgent urinary incontinence (UUI) Called; If both urgency and stress are present together, it is called a hybrid type (MUI) [6, 7].

Urinary incontinence has been identified as a World Health Organization health priority [8]. Urinary incontinence has many physical, mental and social effects on women’s lives [9, 10], common mental problems in these people include anxiety and depression [11, 12]. Physical consequences include pressure sores [12], sleep disturbances and decreased sleep quality [13], urinary tract infections [14], falls and fractures, which are the leading causes of death in people over 65 [15].

Urinary incontinence has a great impact on daily and social activities such as work, travel, physical exercise and sexual function [16, 17] and thus reduces the quality of life [18]. Urgent incontinence is more common in nervous system disorders such as Parkinson’s, multiple sclerosis, and spinal and pelvic nerve damage [19, 20]. Age-related changes in the lower urinary tract include decreased bladder capacity and a feeling of fullness, decreased detrusor muscle contraction rate, decreased pelvic floor muscle strength, and increased residual urine volume [21].

The prevalence of urinary incontinence among older women has been reported in different studies, with an overall prevalence of 14% in US studies [22, 23]. In studies conducted in European countries, the prevalence of urinary incontinence has been estimated at 37% [24, 25]. In studies conducted in different regions of Asia, the prevalence of urinary incontinence in older adults was estimated at 13% [26, 27] and in Africa 45.3% [28]. In the study conducted in Middle Eastern countries, the prevalence of urinary incontinence was reported to be 52% [29–31].

In a study conducted in Iran, in a study in northern Iran (2016), one-third of older adults’ women in the city of Babol had urinary incontinence [32], in a study conducted in Yazd (2015) among women over 60 years, the prevalence of urinary incontinence was 62.2% [31]. Given the different prevalence reported and the need for consistent doses for intervention measures, and given that women cannot avoid aging and childbirth, awareness of the risk factors for urinary incontinence should be promoted.

On the other hand, studies in this field provide opaque and different information and the effective factors affecting urinary incontinence in older adults women in different studies report different reporting amounts and heterogeneity. Therefore, this study aims to answer the questions of the prevalence of urinary incontinence in older women in the world and what are the factors affecting this incontinence?

## Methods

### Registration number

This study has been registered with the code (IR.KUMS.REC.1399.455), in the deputy of research and technology of Kermanshah University of Medical Sciences.

### Search method and time domain

This study is a systematic review and meta-analysis and is the result of extracting the findings of studies conducted in this field. First, articles published in domestic and foreign journals were retrieved by searching in databases, MEDLINE, Cochrane Library, Sciencedirect, Embase, Scopus, ProQuest, and Persian databases including iranmedex, magiran and SID in the period January 2000 to April 2020.

The researcher uses the keywords urinary incontinence, women, the older adults, urinary disorders, or similar words in Persian sources and examines English-language databases using the words: Incontinence, women, older adults, urinary disorders, Prevalence, risk factor Urinary.

Also in the google scholar search engine, both words will be done in Persian and English, and the AND, OR and NOT operators will be used in combination for more comprehensive access to all articles, so the OR pragmatist will be used to check common letters about a disorder such as (Urinary incontinence OR Urinary disorders OR Urinary Reflex Incontinence OR Urinary Urge Incontinence), (Older adults OR Aging).

As well as the word AND among the keywords: (Urinary incontinence AND older adults AND Women) will be used through word matching in the MeSH Browser.

Each article was read by two browsers independently and if the article was rejected, the reason for its rejection was mentioned and in case of disagreement between the two browsers, the article was judged by the third browser and the third referee was considered. Prevalence of study disorder based on PRISMA diagram for entering meta-analysis and to manage articles and remove duplicate articles the EndNote software has been used (version X7, for Windows, Thomson Reuters).

### Selection criteria and entry and exit criteria

Articles in Persian and English are taken from cross-sectional studies as well as case-control articles, all in the group to select the factors affecting urinary incontinence in older adults’ women had the selection criteria to enter the study. And review articles, articles that do not have access to full text despite the relationship with the author of the article and lack of proper response, as well as articles that are of low quality in the evaluation of quality evaluation were removed from the review list.

### Quality assessment and evaluation of the risk of bias

The Newcastle-Ottawa Scale (NOS) is a quality assessment tool for observational studies that are recommended by the Cochrane Collaboration [21]. The NOS assigns up to a maximum of nine points for the least risk of bias in three domains: 1) selection of study groups (four points); 2) comparability of groups (two points); and 3) ascertainment of exposure and outcomes (three points) for case-control and cohort studies, respectively [21], and 11 scores possible. Eventually, articles were classified as high quality (scoring ≥5 points) or low quality (scoring< 5 points). In this meta-analysis, all the articles that obtained five or more points were included.

### Statistical analysis

Data were analysed using Comprehensive Meta-analysis software (Biostat, Englewood, NJ, USA version 3). To evaluate the heterogeneity of selected studies, the I^2^ index test was used. If high heterogeneity is obtained in studies (75% < I^2^), random effects model will be used for meta-analysis of studies, and if low heterogeneity is obtained (I^2^ < 25%), the fixed effects model will be used for the analysis of studies [21]. also, to investigate the publication bias and regarding the high volume of samples included in the study, The Begg and Mazumdar test and its corresponding Funnel plot were used at a significance level of 0.1. the meta-regression test was used to investigate the effects of potential factors influencing the heterogeneity of the studies.

## Results

### Search output

**Fig. 1:**
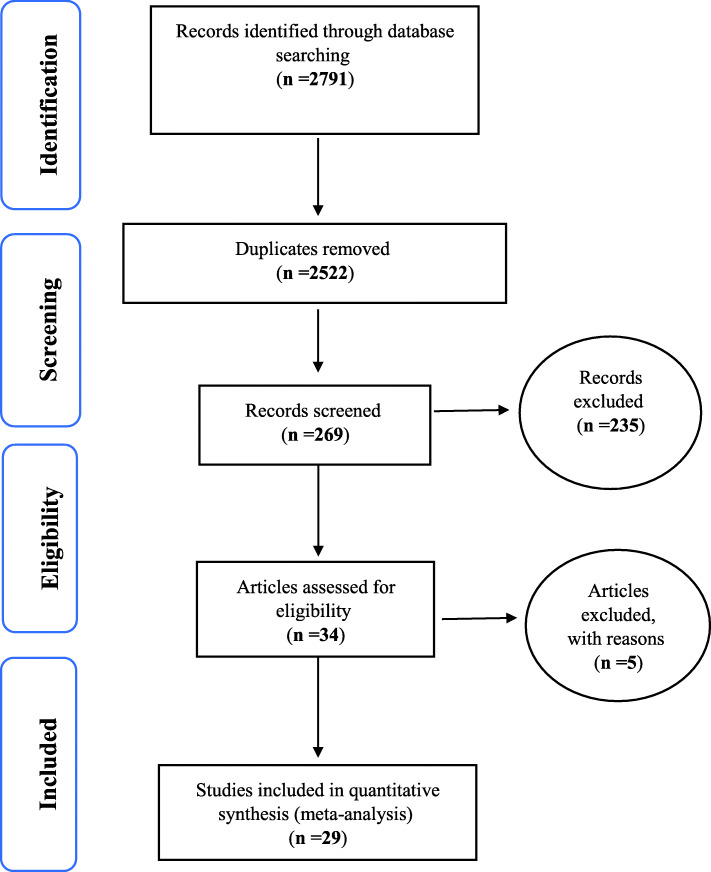
The flowchart on the stages of including the studies in the systematic review and meta-analysis (PRISMA 2009)

Based on studies on the prevalence and factors related to urinary incontinence in older women and including articles published in domestic and foreign journals and search in Cochrane Library Sciencedirect, Embase, Scopus, ProQuest and Persian databases including iranmedex, magiran and SID and in total searches: 2791 items were found. Then, the articles that had the initial conditions for inclusion in the study, based on the initial reviews by deleting 2522 duplicate articles and deleting 235 articles unrelated to the subject of study and deleting 5 articles during the secondary reviews due to lack of access to abstracts and main articles and low quality of articles (This number of deleted items from articles due to lack of access to the full text of articles and their abstracts due to being old or removed from the site of some journals and also their low quality in quality evaluation, of course, the deleted items due to low quality in the study is very limited.). The article entered the meta-analysis process (Fig. 1) (Table 1).

**Table 1:** Specifications of studies entered the study

Row	Author [References]	Publication year	Area	Participants’ Age	Sample size	Prevalence	Quality assessment
1	Ma_gfiret Kaşıkçıa [29]	2015	Turkey	≥65	1094	51.6	Moderate
2	Mary K. Townsend [33]	2017	Mexico	≥60	1289	9.5	Moderate
3	Samreen Khan [30]	2017	India	≥60	149	46.3	Moderate
4	Larissa Pruner Marques [23]	2015	Brazil	≥60	1089	36.3	High
5	E. Moudi [34]	2017	Iran	≥60	590	32.9	High
6	Khanighaleejogh R [35]	2011	Iran	68–84	114	54.2	Moderate
7	David V. Espino [36]	2003	USA	≥65	1589	15	Moderate
8	Stefania Maggi [37]	2001	Italy	≥65	1531	21.6	Moderate
9	Yu Ko [38]	2005	USA	≥65	58,255	27.5	Moderate
10	Jing Ge [39]	2015	China	≥60	627	22.1	High
11	Juliana Schulze Burti [40]	2012	Brazil	≥65	246	50	Moderate
12	Rochani Sumardi [41]	2014	Indonesia	≥60	273	24.2	Moderate
13	Gileard G. Masenga [42]	2019	Tanzania	55–90	274	48.5	Moderate
14	Jennifer M. Wu [43]	2015	USA	≥60	2423	20.6	Moderate
15	Mary K. Townsend [33]	2017	Mexico	≥60	1168	10.3	Moderate
16	Lei Zhang [44]	2014	China	≥60	3753	51.6	Moderate
17	Jarosław Pinkas [45]	2016	Poland	90–106	870	60	High
18	Javier Jerez-Roig [46]	2016	Brazil	≥60	240	40.8	Moderate
19	Renata B. Reigota [47]	2016	Brazil	≥60	379	53.6	Moderate
20	Nazli Sensoy [48]	2013	Turkey	≥60	203	29.3	High
21	J. Marleen Linde [49]	2017	Netherlands	≥60	189	56.6	Moderate
22	Walaa W. Aly [50]	2020	Egypt	≥60	130	80	Moderate
23	Prabhu, Shruti Atul [51]	2013	India	≥60	58	41.1	High
24	Bo Liu [52]	2014	China	≥60	1417	54.2	Moderate
25	Pamela L [53]	2013	Canada	≥65	331,000	14	High
26	Rui Luo [54]	2017	Singapore	≥60	22	59.09	High
27	Catherine A. Matthews [55]	2013	America	62–87	64,396	38	Moderate
28	Lea F. Schumpf [56]	2017	Switzerland	≥65	44,811	54.7	Moderate
29	Olga NTkacheva [57]	2018	Russia	≥65	286	40.2	High

### Review of publication bias and meta-analysis

**Fig. 2:**
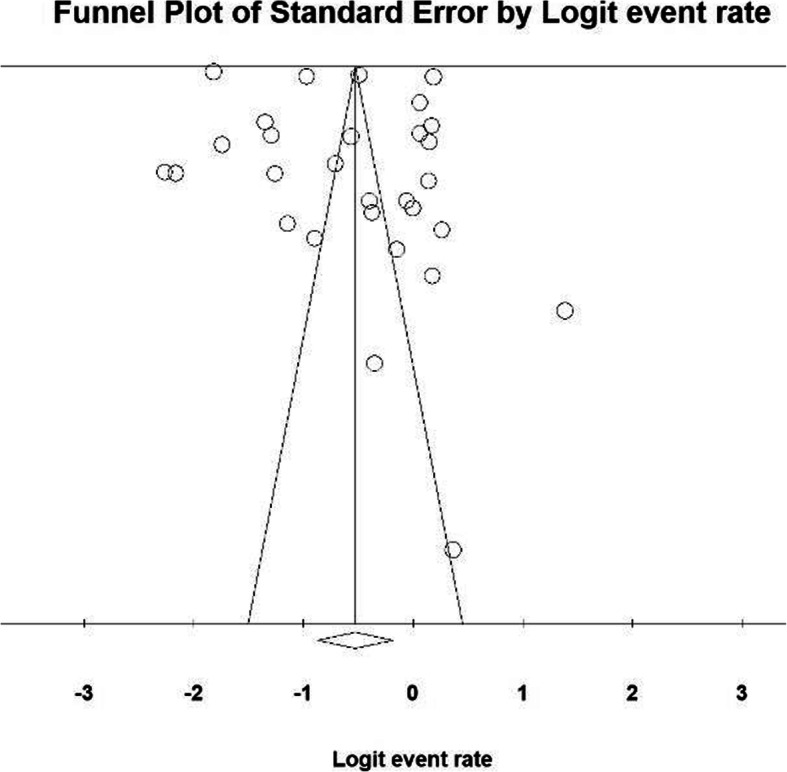
Funnel Plot Results of urinary incontinence in older adults’ women worldwide

The heterogeneity of the studies was investigated using the I^2^ test and based on this test, the amount of heterogeneity (I^2^ = 99.9%) was obtained and shows high heterogeneity in the included studies, so the random-effects model was used to combine the results of the studies. Also, the results of the study of publication bias in the studies were evaluated due to the high sample size entered in the studies with Begg and Manzumdar test and with a significance level of 0.1, which indicates that the diffusion bias was not significant in the present study (*P* = 0.252) (Fig. 2).

**Fig. 3:**
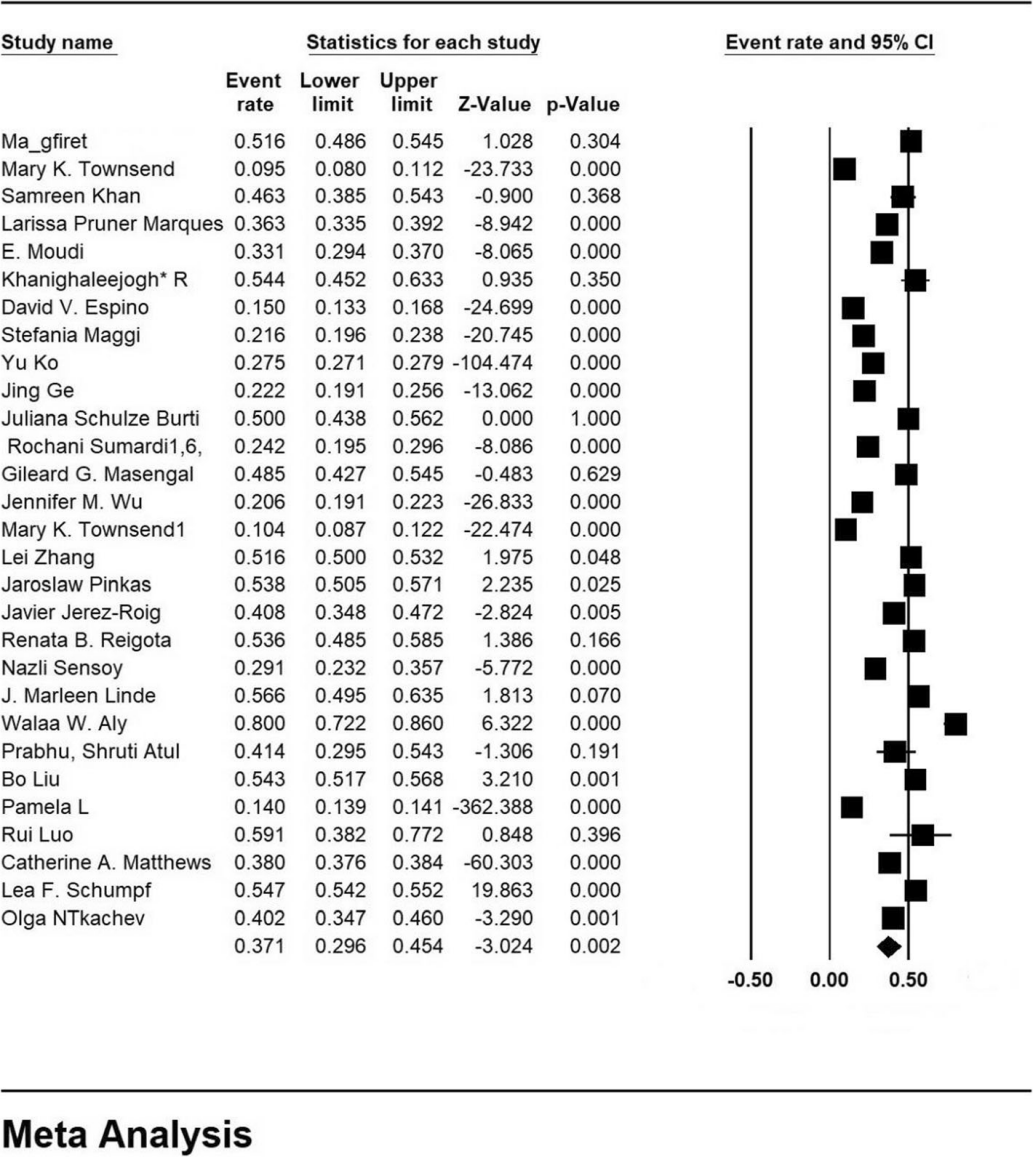
Overall prevalence of urinary incontinence in older adults’ women worldwide based on a random effects model

A review of 29 studies and the sample size of 518,465 people in the age range of 55–106 years, urinary incontinence in the older adults’ women of the world based on a meta-analysis of 37.1% (95% CI: 29.6–45.4%) was obtained. The highest prevalence of urinary incontinence in older adults’ women in Egypt with 80% (95% CI:72.2–86%) in 2020 [50] and the lowest prevalence of urinary incontinence in older adults’ women in Mexico with 9.5% (95% CI:8–11.2%) was achieved in 2017 [33] (Fig. 3).

In this figure, the prevalence of urinary incontinence is shown based on the random-effects model, in which the black square, the colour of the prevalence, and the length of the line segment on which the square is placed are 95% confidence intervals in each study.

### Sensitivity analysis

**Fig. 4:**
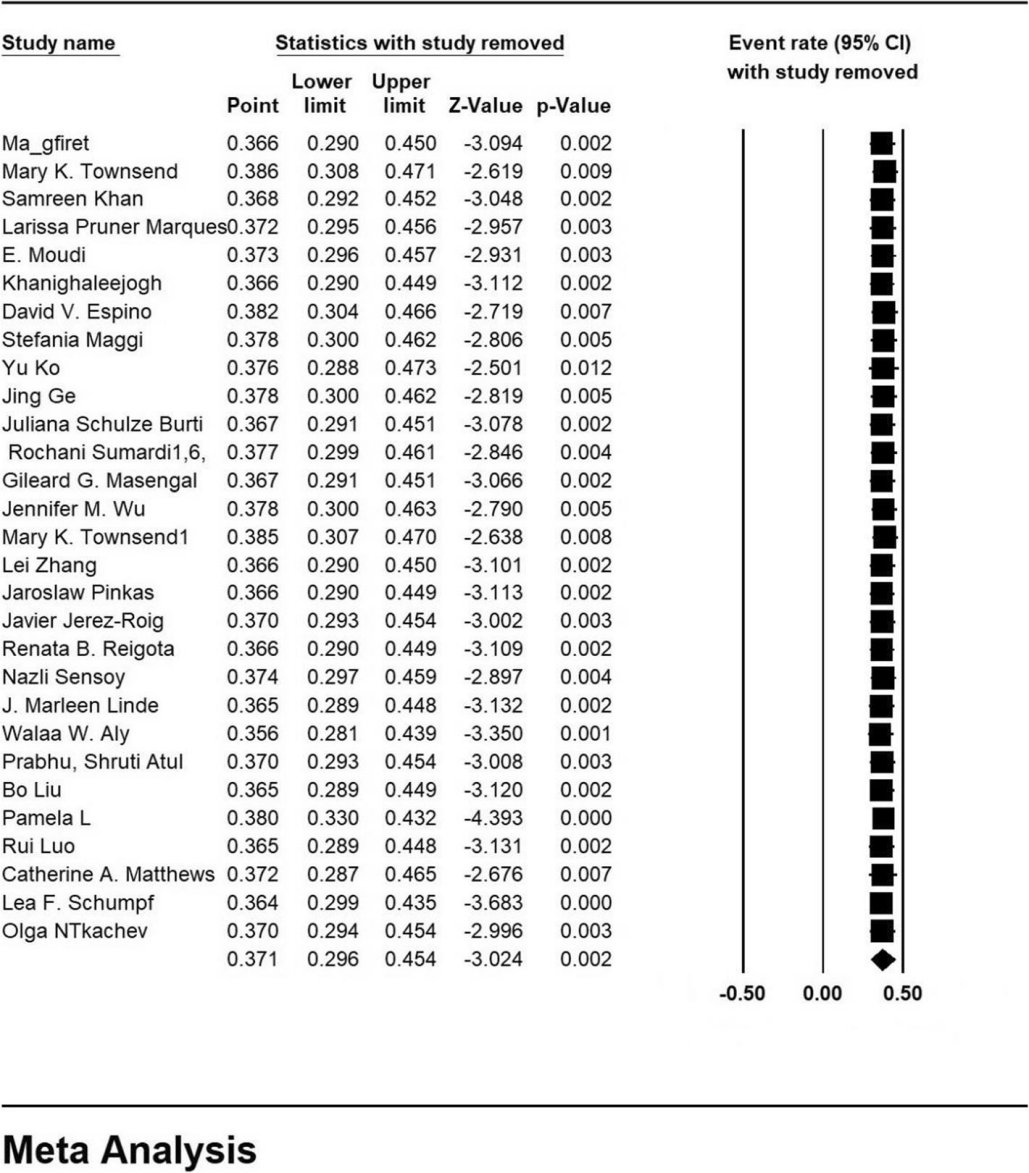
Results of sensitivity analysis

A sensitivity analysis was perfumed to ensure the stability results, after removing each study results did not change (Fig. 4).

### Meta-regression test

**Fig. 5:**
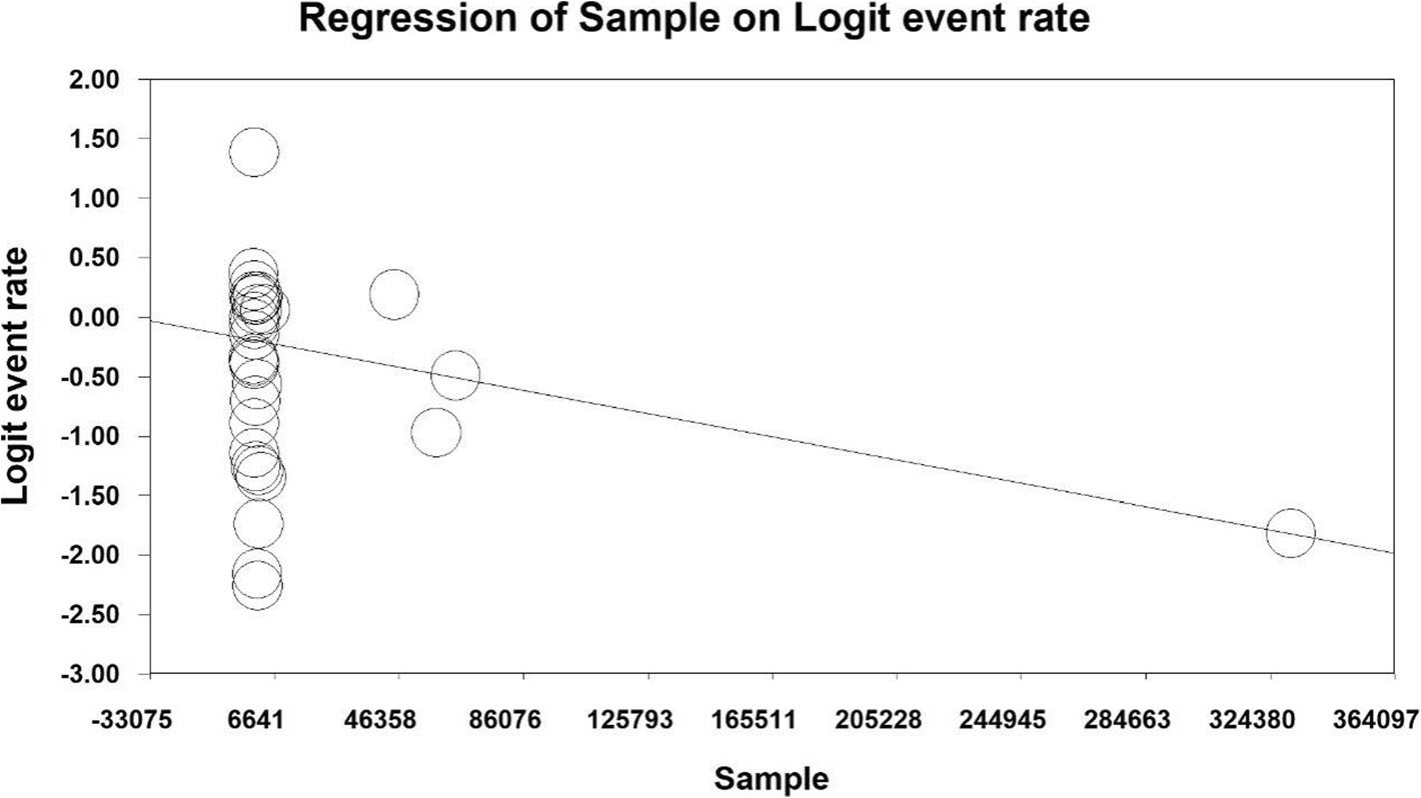
Meta-regression diagram of the overall prevalence of urinary incontinence in older adults’ women worldwide by sample size

**Fig. 6:**
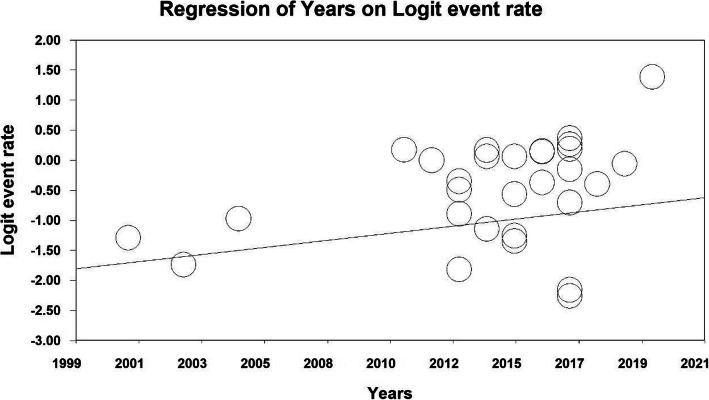
Meta-regression diagram of the overall prevalence of urinary incontinence in older adults’ women worldwide by year of release

To investigate the effects of potential factors influencing the heterogeneity of the overall prevalence of urinary incontinence in older women around the world, meta-regression was used for two factors: sample size and year of study (Figs. 5 and 6). According to Fig. 5, with increasing sample size, the overall prevalence of urinary incontinence in the older adults omen of the world decreases (*P* <  0.05). It was also reported in Fig. 6 that with increasing the year of the study, the overall prevalence of urinary incontinence in the older adults women of the world increases (*P* <  0.05).

### Subgroup analysis by continent

Based on the results of Table 2, the highest prevalence of urinary incontinence in older adults women was reported in Asia with 45.1% (95% CI: 36.9–53.5%). The results of this table also report that no diffuse bias was observed in the study by continent, and the study of metallic mercury was also reported in each continent.

**Table 2:** Evaluation of urinary incontinence in older adults’ women by different continents

continents	Number of articles	Sample Size	I^**2**^	Begg and Mazumdar Test	Prevalence % (95% CI)	Meta-regression		***p*** value
						Samples	Years	
Asia	12	7419	97.1	0.394	45.1 (95% CI: 36.9–53.5)	increase	decrease	< 0.05
Europe	6	48,698	99.1	0.247	43.8 (95% CI: 32.2–56.1)	increase	increase	< 0.05
America	11	462,074	99.9	0.535	25.8 (95% CI: 18.2–35.3)	decrease	decrease	< 0.05

### Effective and related factors in urinary incontinence in older adults’ women

According to a systematic review of studies, various factors affect the incidence of urinary incontinence in older women, the most important of which are the age of women [25, 26, 38, 49, 50, 58–64], obesity based on BMI index [25, 37, 48, 49, 52, 58, 59, 62, 63, 65–67], diabetes [25, 26, 37, 49, 52, 58, 62, 66–68], women’s education [26, 30, 36, 48, 52, 58, 61], delivery rate [23, 37, 59, 60, 62, 67], hypertension [26, 66, 67], smoking [30, 36, 37, 52, 60, 62] as well as urinary tract infections [23, 49, 52]. Based on the results reported in Table 3, all these factors have a significant difference in the incidence of urinary incontinence in older adults’ women (*p* <  0.05).

**Table 3:** A systematic review of the factors affecting older adults’ women with urinary incontinence

Author [References]	Place of study	type of study	Risk factors examined	*p*-value
S.A. Eshkoor 2017 [27]	Malaysia	Case-control	Blood Triglycerides	0.015
			Albumin	0.026
			HDL	0.029
			Monounsaturated fat	0.009
			Cataract-glaucoma	0.051
			Tiredness	0.039
			Constipation	< 0.001
			Gastric-Ulcer Problem	< 0.001
			Vision-hearing loss	0.010
			Joint pain	0.002
Shi LU et al. 2016 [65]	China	Cross-sectional	Age	0.041
			BMI	0.027
			Menstrual status	0.036
			Mode of delivery	0.007
			Heart disease	0.02
			Dyslipidemia	0.038
			Arthritis	0.003
			Gynecological disease	< 0.001
			Chronic pelvic pain	< 0.001
			Atrophic vaginitis	< 0.001
			Constipation	< 0.001
			Fecal incontinence	< 0.001
Ralf Suhr et al. 2017 [25]	Germany	Cross-sectional	Musculoskeletal disease	0.002
			Stroke	0.035
			Cancer	0.003
			Dementia	< 0.001
			Live with barriers	0.129
			Living alone	0.143
			BMI	0.01
			Age	0.06
			Female sex	0.007
			Respiratory	0.158
			Diabetes	0.798
			Cardiovascular	0.002
			Psychiatric	0.927
Pedersen et al. 2017 [58]	Germany and Denmark	Analytical descriptive	Age	< 0.001
			BMI	0.001
			Diabetes	0.007
			Chronic obstructive pulmonary disease	0.002
			Vaginal deliveries	< 0.001
Ma_gfiret Kaşıkçı et al. 2015 [36]	Turkey	Cross-sectional	BMI	< 0.001
			Smoking	0. 047
			Constipation	< 0.001
			Urinary tract infection	< 0.001
			Chronic diseases	< 0.001
			Familiar history	< 0.001
			Complaint of chronic coughing	0. 530
			Hormone replacement	< 0.001
			Genital prolapse	< 0.001
			Cystocele	< 0.001
			Urogenital operation	< 0.001
			Nocturia	< 0.001
Kyungjin Sohn et al. 2018 [26]	Korea	Longitudinal Study	Age	< 0.001
			Education	< 0.001
			Marital status	0.043
			Chronic lung disease	0.034
			Cerebrovascular disease ΙΙ	0.002
			Social activity	0.007
			Arthritis	< 0.001
			Difficulty in daily living due to visual problems	< 0.001
			Difficulty in daily living due to hearing problems	< 0.001
			Experience of fall in the last 2 years	0.017
			Psychiatric disease	0.008
			Fear of falling	< 0.001
			Psychiatric disease	0.008
Samreen Khan et al. 2017 [30]	India	Cross-sectional	Years spent in menopause	0.002
			parity	0.001
			Hysterectomy	0.006
			UTI	< 0.001
			Pelvic organ prolapse	0.031
Sanae Ninomiya et al. 2017 [59]	Japan	Cross-sectional	Age	< 0.001
			BMI	< 0.001
			parity	0.009
			Mode of delivery	< 0.001
			Constipation	0.01
Larissa Pruner Marques et al 2015 [23]	Brazil	Cross-sectional	Gender	< 0.001
			Age	< 0.001
			Education	< 0.001
			Physical activity	< 0.001
			Dependence	< 0.001
			Cognitive deficiency	< 0.001
			Depressive symptoms	< 0.001
			Diabetes	< 0.001
			Bronchitis or asthma	< 0.001
			Hypertension	< 0.001
			Cardiovascular	< 0.001
			Stroke	< 0.001
			Nutritional state	0.017
			Polypharmacy	< 0.001
			Self-rated health	< 0.001
E. Moudi et al. 2017 [34]	Iran	Cross-sectional	Marital status	0.03
			Constipation	0.01
			Steroid drug	0.04
David V. Espino et al. 2003 [36]	Mexico	Cross-sectional	Education	0.03
			BMI	0.03
			Diabetes	0.01
			Smoking	< 0.001
			Impaired activities of daily living	0.03
			Age	0.02
Stefania Maggi et al. 2001 [37]	Italy	Cross-sectional	Age	< 0.001
			Marital status	< 0.001
			Education	< 0.001
			Mental Health	< 0.001
			Depression	0.028
			Mobility disability	< 0.001
			ADL disability	< 0.001
			BMI	< 0.001
			Smoking	< 0.001
			Self-rated health	< 0.001
Marit Helen Ebbesen et al. 2013 [60]	Norway	Cross-sectional	Age	< 0.001
			BMI	< 0.001
			Self-perceived health status	< 0.001
			Smoking	0.009
			Alcohol	0.016
			Parity	< 0.001
			Diabetes	0.029
			Angina	0.021
			Heart attack	0.047
			Stroke	0.032
Clemens Wehrberger et al. 2012 [68]	Austria	longitudinal, population-based study	Alzheimer	0.073
Jeongok Park et al. 2015 [66]	Korea	Analytical descriptive	Age	< 0.001
			BMI	0.02
			Place of residence	0.003
			Self-reported health status	< 0.001
			Hypertension	< 0.001
			Stroke	< 0.001
			Diabetes	< 0.001
			Asthma	< 0.001
			Depress	< 0.001
			Falls	< 0.001
			Functional ability	< 0.001
			Physical strength	< 0.001
Jing Ge et al. 2015 [39]	China	Analytical descriptive	Age	< 0.001
			Job	< 0.001
			Education	< 0.001
			BMI	< 0.001
			Income/month	0.014
			Smoking	0.023
			Physical exercise frequency	< 0.001
			Menstrual status	< 0.001
			Pregnancy history	< 0.001
			Abortion times	< 0.001
			Parity	< 0.001
			Age at first delivery	< 0.001
			Mode of delivery	< 0.001
			Chronic pelvic pain	< 0.001
			Respiratory disease	< 0.001
			Digestive disease	< 0.001
			Cardiovascular	< 0.001
			Neurologic disease	0.003
			Osteoarticular disease	< 0.001
			Hyperlipemia	< 0.001
			Diabetes	< 0.001
			History of pelvic surgery	< 0.001
			Gynecological disease	< 0.001
			Constipation	< 0.001
			Fecal incontinence	< 0.001
Juliana Schulze Burti et 2012 [40]	Brazil	Cross-sectional	Diabetes	0.022
			hypertension	0.008
Joshua A. Cohn et al. 2018 [61]	USA	Cohort	Age	< 0.001
			Education	0.034
Vatche A. Minassian et al. 2020 [62]	USA	Cohort	Age	< 0.001
			BMI	< 0.001
			Parity	< 0.001
			Smoking	< 0.001
			Physical activity	< 0.001
			Diabetes	< 0.001
			History of vascular disease	< 0.001
			Postmenopausal hormone use	< 0.001
			Baseline UI severity	< 0.001
MáyraCeciliaDellú et al. 2016 [63]	Brazil	Cross-sectional	Pregnancy	< 0.001
			Post-partum	< 0.001
			Genital prolapse	< 0.001
			Stress	< 0.001
			Depression	< 0.001
			BMI	< 0.001
Javier Jerez-Roig et al. 2016 [46]	Brazil	Cross-sectional	Ethnicity	0.005
			Stroke	0.003
			Physical activity	0.03
Ramazan Altintas et al. 2013 [67]	Turkey	Retrospective study	Age	< 0.001
			BMI	< 0.001
			Parity	< 0.001
			hypertension	0.008
			Diabetes	< 0.001
			Birth trauma	< 0.001
			Gynecological surgery	< 0.001
Nazli Sensoy et al. 2013 [48]	Turkey	Cross-sectional	Age	< 0.001
			Marital status	< 0.001
			Education	< 0.001
			Job	< 0.001
			BMI	< 0.001
			*Number of Deliveries*	< 0.001
			*Episiotomy*	< 0.001
			*Abortion*	< 0.001
			*Age at first delivery*	< 0.001
			*4 kg baby delivered*	< 0.001
J. Marleen Linde et al. 2017 [49]	Netherlands	Cross-sectional	Age	< 0.001
			BMI	< 0.001
			UTI	< 0.001
			Nocturia	0.04
			Fecal incontinence	0.004
			Constipation	< 0.001
			Diabetes	< 0.001
			Vaginal hysterectomy	< 0.001
			Childbirth history	< 0.001
			Number of deliveries	< 0.001
Bo Liu et al. 2014 [52]	China	Cross-sectional	BMI	< 0.001
			Monthly Income	< 0.001
			Education	< 0.001
			Residence	< 0.001
			Physical activity	< 0.001
			Labor	< 0.001
			Physical activity	< 0.001
			Hyperlipemia	< 0.001
			Cardiovascular	< 0.001
			Nervous System Disease	< 0.001
			Diabetes	< 0.001
			Nocturia	< 0.001
			Constipation	< 0.001
			Alcohol	< 0.001
			Smoking	< 0.001
			Prolonged Labor	< 0.001
			Chronic pelvic pain	< 0.001
			Marital status	< 0.001
			Respiratory disease	< 0.001
			Pregnancy	< 0.001
			UTI	< 0.001
			Mode of delivery	< 0.001
Walaa W. Aly et al. 2020 [50]	Egypt	Cross-sectional	Praying	< 0.001
			Social activities	< 0.001
			Physical recreational activities	0.002
			Anxiety	< 0.001
			Depression/hopelessness	< 0.001

## Discussion

Urinary incontinence is a very common condition that usually increases with age in women. Having general information about the prevalence of this disorder and identifying risk factors is useful and even necessary that can play an effective role in improving the quality of life and general health of society [4, 57]. This meta-analysis study was performed on 518,465 older adults women and the prevalence of urinary incontinence in older adults women was 37.1%. However, in the study of the prevalence of incontinence in older adults women by continents, the highest prevalence of urinary incontinence was reported in older adults women in Asia with 45.1%.

In a study conducted in Egypt (2020), the prevalence of incontinence among older women was 80% [50]. In the study of Summer Khan et al. in India (2018) the overall prevalence of urinary incontinence was 46.3% [30], in a study in Russia (2018) the prevalence of incontinence in older adults women was 40.2 [57].

In a study conducted in Iran (2017), it was reported that one-third of older women (33%) have urinary incontinence [34]. In another study conducted in Iran as a systematic review and meta-analysis (2018), the overall prevalence of urinary incontinence in women was estimated at 46% [64].

Based on the results, the highest prevalence of urinary incontinence in older adults’ women was reported in Asia with 45.1% and the lowest prevalence of urinary incontinence in older adults’ women was reported in America with 25.8%, By observing the prevalence in different regions, it can be concluded that the prevalence of urinary incontinence in different populations is completely different, which can be due to differences in culture or tools and methods of study.

It can also show the effect of ethnoreligious factor on the insignificance of urinary incontinence in older adults’ women in Asian countries, this issue has been stated and reported in the study of Touhidi Nezhad and et al. this study is about rectovaginal fistula and explains the importance and says that The rectovaginal fistula is a complex and multifaceted problem with social, individual, familial, religious, and ethnic-environmental dimensions [69], this can embarrass Asian women and hide and increase the prevalence of urinary incontinence in older women.

The high prevalence obtained in this study shows the need to investigate and follow up this condition, due to the significant impact of this disorder on depression and quality of life of older adults’ women, requires special attention and screening for urinary incontinence in treatment and care programs in the country. Various studies have mentioned various factors in the incidence of urinary incontinence in women, such as age, menopause, delivery and number of deliveries, obesity, and diabetes are among the most important of these factors [25, 70].

Age is one of the important factors in the prevalence of urinary incontinence. Changes related to aging in the lower urinary system include: decreased bladder capacity and feeling of fullness, decreased rate of detrusor muscle contraction, decreased pelvic floor muscle resistance and increased residual urine volume [21].

In a study by Marland Lind et al. in the Netherlands and a study by Nazli et al. in Turkey, aging was one of the most influential factors in urinary incontinence [48, 49], while in a study in Brazil [46] In Iranian older adults women, no relationship was observed between urinary incontinence and aging [34]. Menopause, with a decrease in estrogen and a decrease in collagen, reduces the elasticity of the detrusor muscle of the ductus arteriosus and atrophic changes in the pelvic floor muscles and increases urinary incontinence in women [71].

In the study conducted in Turkey, menopause is one of the most important factors influencing female incontinence [48], while in the study of Aquarius et al. in Brazil, no significant relationship was reported between menopause and the increased prevalence of urinary incontinence [72]. Urinary incontinence is higher in women with more deliveries and vaginal deliveries. These two factors seem to be one of the most important risk factors for urinary incontinence in women [73]. In the study conducted among Chinese women, there is a type of delivery and the possibility of urinary incontinence [52], also in the study of Marland Lind et al. there was a significant relationship between delivery history, number and type of delivery with increased urinary incontinence [49]. However, in a study in India, no association was found between childbirth and urinary incontinence [30].

Obesity is an exacerbating condition of urinary incontinence, which can be caused by the accumulation of excess weight on the urinary tract during life [22]. Many studies have shown an association between obesity and increased urinary incontinence. In a study by Ninomia et al. in Japan [59] and a study by Hong et al. in the United States [74], a significant relationship was found between weight gain and increased incidence of urinary incontinence.

Also, the level of education is considered as one of the components of individual and social development and its role in personal health and also a factor in increasing the quality of life [9]. In his study by Espanyo et al. in Mexico and the United States [36] and in the study by Marcos et al. in Brazil [23], increasing the level of education was reported to be an important factor in reducing the incidence of urinary incontinence. No urinary incontinence was reported between education levels [66].

Diabetes can cause UI by several mechanisms, hyperglycaemia causes increased urine volume and increased activity of the bladder muscle, and ultimately causes dysfunction of this muscle. Diabetic cytopathic and bladder nerve damage are other effective complications [75]. In a study by Absen et al. in Norway, it was reported that there was a significant association between diabetes and urinary incontinence [60], while a German study found no association between diabetes and urinary incontinence [25].

Chronic respiratory diseases are associated with symptoms such as a cough that can cause urinary incontinence [76]. In a study based on the population of Jinge Ge et al. in China, a significant relationship was reported between lung disease and incidence [39]. However, in the study of Ralph Souher et al. in Germany [4] and the study of Sohan et al. in Korea [26], no significant relationship was observed between urinary incontinence and respiratory disease.

Nervous system disorders are seen as an important factor in the prevalence of urgent incontinence [19, 20]. There were mental illnesses, cancer and conditions such as living alone [25]. A study by Kasik et al. in Turkey also reported obesity, smoking, a history of constipation, UTI, family history, chronic illness, chronic cough, a history of hormone therapy, genital prolapse, a history of urology, and a history of communication impairment. Have significance with incontinence [46].

In a promising study by colleagues in Iran, it was reported that urinary incontinence is directly and significantly related to factors such as marital status, constipation, and corticosteroid medications, while urinary incontinence is associated with factors such as age, obesity, education, number of children, diabetes, hypertension, and Respiratory disorders were not associated [34].

In a 2016 study by Aquarius et al. in Brazil, the factors that increased urinary incontinence in women included: number of pregnancies, deliveries, genital prolapse, anxiety, depression, and obesity [72]. In a study by Marcos et al. in Brazil, there was a significant relationship between age, education, physical activity, dependence, cognitive problems, symptoms of anomia, bronchitis, asthma, cardiovascular disease, diabetes, hypertension, stroke and ischemia, nutritional status, polypharmacy, self-Urinary incontinence was reported [23].

Given the above, it is necessary for physicians and specialists to consider adults’ women in the age group of 55 to 106 years according to the criteria recommended by the International Continence Society (ICS) and to standardize the criteria so that diagnostic and treatment strategies are more effective.

### Limitations

The most important limitations of the present study are the high heterogeneity of studies, which can be due to sampling size, age groups, geographical areas, races, and other different factors in the studies, which can be controversial in the study.

## Conclusion

Given the high prevalence of urinary incontinence in older women around the world, health policy makers must considerand diagnostic measures in older women and prioritize treatment and rehabilitation activities.

## Data Availability

Datasets are available through the corresponding author upon reasonable request.

## Abbreviations

WHO: World Health Organization
ICS: International Association of Urinary Incontinence
UI: urinary incontinence
SUI: Stress urinary incontinence
UUI: Urgent urinary incontinence
NOS: The Newcastle-Ottawa Scale
SID: Scientific information database
PRISMA: Preferred reporting items for systematic reviews and meta-analysis
STROBE: Strengthening the reporting of observational studies in epidemiology for cross- sectional study
