# Correction: Prevalence and factors related to urinary incontinence in older adults women worldwide: a comprehensive systematic review and meta-analysis of observational studies

**DOI:** 10.1186/s12877-022-03111-6

**Published:** 2022-05-25

**Authors:** Sedighe Batmani, Rostam Jalali, Masoud Mohammadi, Shadi Bokaee

**Affiliations:** 1 Department of Nursing, School of Nursing and Midwifery, Kermanshah University of Medical Sciences, Kermanshah, Iran; 2 Faculty of Health and Life Sciences, School of Life Sciences, Coventry University, Coventry, UK

**Correction to: BMC Geriatr 21, 212 (2021)**

**https://doi.org/10.1186/s12877-021-02135-8**

After publication of this article [1], it was reported that in this article in reference [3], the author name “Pierre EF” was incorrectly written as “Fiore PE”.

The original article [1] has been updated.
